# Cytokine secretion patterns distinguish herpes simplex virus type 2 meningitis from herpes simplex virus type 2 genital herpes

**DOI:** 10.3389/fimmu.2025.1515741

**Published:** 2025-06-04

**Authors:** Moa Bjerhem, Alexandra Svensson, Marie Studahl, Petra Tunbäck, Eva-Marie Boman, Kenny Brandström, Christine Lingblom, Azadeh Reyahi, Karolina Thörn, Kristina Eriksson

**Affiliations:** ^1^ Department of Rheumatology and Inflammation Research, Institute of Medicine, University of Gothenburg, Gothenburg, Sweden; ^2^ Department of Dermatovenerology, Sahlgrenska University Hospital, Gothenburg, Sweden; ^3^ Department of Infectious Diseases, Institute of Biomedicine, University of Gothenburg, Gothenburg, Sweden; ^4^ Department of Infectious Diseases, Sahlgrenska University Hospital, Gothenburg, Sweden; ^5^ Department of Dermatovenerology, Institute of Clinical Sciences, University of Gothenburg, Gothenburg, Sweden; ^6^ Department of Infectious Diseases, Södra Älvsborg Hospital, Borås, Sweden; ^7^ Department of Infectious Diseases, Skaraborg Hospital, Skövde, Sweden; ^8^ Department of Clinical Microbiology, Sahlgrenska University Hospital, Gothenburg, Sweden

**Keywords:** HSV-2, meningitis, genital herpes, cytokines, multivariate analysis, immune profile

## Abstract

The aim of this study was to identify immune factors that distinguish patients with herpes simplex virus type 2 (HSV-2) meningitis from patients with HSV-2 genital herpes by analyzing demographic data, *in vitro* production of cytokines and other immune factors secreted by patient peripheral blood mononuclear cells (PBMC), and existing antibody responses. PBMC and plasma were collected from patients previously diagnosed with HSV-2 meningitis (n=49) and HSV-2 genital herpes (n=38). PBMC were cultured in the presence or absence of HSV-2 and followed by multiplex analyses of culture supernatants for a panel of immune factors including Th1 and inflammatory cytokines, interferons, and chemokines. Plasma was analyzed for type-specific HSV antibodies and HSV-2 DNA. The multivariate method OPLS-DA was used to identify immune response patterns that differentiate the two patient groups. The multivariate analysis showed that the immune profile differed significantly between the two different HSV-2 disease manifestations. Meningitis patients were distinguished by the spontaneous production of several anti-viral immune factors by PBMC including type I and type III IFNs. PBMC from HSV-2 meningitis patients also secreted significantly higher levels of IFN-γ in response to HSV-2 compared to PBMC from HSV-2 genital herpes patients. Blocking the type I IFN receptor reduced the production of HSV-2-induced IFN-γ by PBMC suggesting that enhanced production of type I IFNs could promote IFN-γ recall responses. The levels of HSV-2 type-specific antibodies did not differ between the patient groups. In conclusion, we show that HSV-2 meningitis leads to a more profound activation of both innate and acquired PBMC immune responses, compared to that of HSV-2 genital herpes. Whether these differences are the cause, or the consequence, of the different disease manifestations remains to be determined.

## 1 Introduction

Herpes simplex virus type 2 (HSV-2) is a sexually transmitted human pathogen which gives rise to a chronic (life-long) infection. The virus initially infects epithelial cells in the genital tract and then spreads via sensory nerve endings to the innervating ganglia where a chronic, slowly replicating, infection is established, leading to the release of infectious virus in the genital tract (1). The infection is prevalent with more than 500 million humans infected around the world and an estimated global incidence of 23.6 million new cases annually (2). In Sweden, approximately 15% of adults carry the virus, and HSV-2 is one of the major causes of aseptic meningitis comprising up to 20% of consecutive cases (3, 4).

HSV-2 can present itself clinically in several different ways (1); either not at all (subclinical or asymptomatic infection), as genital herpes, or as meningitis. About, twenty-five to 30% of those infected are asymptomatic and never experience any symptoms of disease. Approximately 70% of infected adults develop genital herpes, which is the hallmark of the infection and is characterized by intermittently recurring ulcers and blisters, preferentially in the genital tract. In some cases, the virus also spread further into the CNS through the meninges causing aseptic meningitis, presenting with or without mucocutaneous blisters and leading to persisting sequela for more than 6 months in 10% of cases (5).

A mechanistic explanation why HSV-2 infection in rare cases cause meningitis is currently lacking. It is known that HSV-1-infected individuals have a partly cross-reactive immune response to HSV-2. This cross-reactive immune response does not protect against HSV-2 infection, but it is associated with asymptomatic seroconversion and milder HSV-2 disease (6, 7). T-cells and perhaps also IFN-γ influence the disease outcomes. T-cell deficiency leads to more aggressive and frequent HSV-2 disease symptoms (1), and HSV-2-specific IFN-γ production is higher in patients with HSV-2 meningitis compared to patients with genital herpes (8). The same holds true for those with an asymptomatic HSV-2 infection (9). HSV-2 meningitis has also been associated with certain HLA-types, low serum concentrations of IgG1 and polymorphisms in the gene encoding mannan-binding lectin (10, 11). On the individual level, exome sequencing of meningitis patients has identified rare mutations in genes encoding proteins involved in autophagy, type I interferon responses, ubiquitin-proteasome pathways, and cell proliferation/apoptosis (12–14) implying an important role of anti-viral innate immune responses and intrinsic cellular functions in the development of meningitis.

To test the hypothesis that HSV-2 meningitis is associated with alterations in the immune system, we set out to identify immune factors that distinguish patients with HSV-2 meningitis from patients with HSV-2 genital herpes by analyzing *in vitro* production of cytokines and other immune factors secreted by patient peripheral blood mononuclear cells (PBMC), and existing antibody responses.

## 2 Materials and methods

### 2.1 Human sample collection

Eighty-eight patients diagnosed with HSV-2 infection were recruited at the STD clinic at Sahlgrenska University Hospital and Infectious Disease clinics at Sahlgrenska University Hospital, Skaraborg Hospital and Södra Älvsborg Hospital. A diagnosis of HSV-2 meningitis required symptoms of viral meningitis and CSF pleocytosis levels above 5 X 10^6^ leukocytes/L with an HSV-2 infection etiology. The etiology was verified by (1) detection of HSV-2 DNA in the CSF by PCR, (2) preceding or concurrent virologically verified HSV-2 lesions in the genital or lumbosacral region, or (3) a history of previous aseptic meningitis of herpetic or unknown origin and positive HSV-2 serology in the acute phase serum sample. For criteria 2 and 3, negative results in bacterial culture and PCR for enterovirus in CSF and negative serology results for tick-borne encephalitis virus were compulsory. A diagnosis of HSV-2 genital herpes required symptoms of genital herpes and an HSV-2 infection etiology confirmed by HSV-2 type-specific PCR. All patients were enrolled either during remission or while on prophylactic anti-viral treatment. All patients donated 20 ml of blood collected in heparin tubes. Permission for this study was granted by the Ethics committee of University of Gothenburg, and all patients gave informed consent. The patients were divided into two groups based on their clinical status, HSV-2 meningitis or HSV-2 genital herpes only (**Table 1**).

**Table 1:** Demographic data of the study cohort.

Demographic Data	All (n=88)	Genital herpes (n=40)	Meningitis (n=48)	P-value
Age at sampling, median (range)	48 (19–82)	39 (22–82)	49 (19–76)	<0.01^B^
Age at diagnosis^A^, median (range)	34 (17–81)	29 (18–81)	37 (17–64)	<0.01^B^
Years between diagnosis and sampling^A^, median (range)	9 (0–32)	6 (0–31)	10.5 (0–32)	>0.05^B^
Female, n (%)	64 (73%)	25 (62%)	39 (81%)	>0.05^C^
Meningitis, n (%)	48 (55%)	0 (0)	48 (100%)	<0.001^C^
Genital/sacral blisters^A^, n (%)	60^A^ (75%)	40 (100%)	20^A^ (50%)	<0.001^C^
HSV-1 positive, n (%)	30 (34%)	16 (40%)	14 (29%)	>0.05^C^
Prophylactic treatment, n (%)	17 (19%)	11 (27%)	6 (12%)	>0.05^C^
Annual episodes of disease, median (range)	–	6 (1–24)	–	
Total episodes of disease, median (range)	–	–	2 (1–10)	

^A^Data missing for 8 patients.

^B^Statistical difference between patients with genital herpes and patients with meningitis (Mann Whitney).

^C^Statistical difference between patients with genital herpes and patients with meningitis (Fisher’s exact test).

### 2.2 Serological analyses

Levels of HSV-2 glycoprotein mgG2-specific antibodies were determined in plasma with a semi-quantitative routine analysis (LIAISON HSV-2 IgG; Diasorin) at the Clinical Microbiology unit, Sahlgrenska University hospital. Co-infection with HSV-1 was assessed using an HSV-1 ELISA kit (HerpesSelect1 ELISA IgG; Focus Technologies). DNA was purified from plasma using Qiagen DNeasy Blood and Tissue Kit (Qiagen) and HSV-2 DNA was detected using qPCR with the HSV-2 TaqMan probe Vi04646232_s1 and TaqMan™ Universal PCR Master Mix (ThermoFisher) for 40 cycles.

### 2.3 Virus strains

The lab-adapted strain HSV-2–333 was obtained as described (15). For antigen preparation, virus was inactivated with UV-light for 30 minutes.

### 2.4 Production of immune factors by human PBMC

Freshly isolated PBMC from HSV-2-infected patients (1x10^6^ cells/ml) were cultured in 96-well plates in a total volume of 200 µl x-vivo medium (Lonza, Verviers, Belgium) supplemented with 1% L-glutamine in the presence or absence of HSV-2 or UV-inactivated HSV-2 (corresponding to 4x10^5^ p.f.u/ml). Culture supernatants were collected after 48 hours and stored at -20 C until assayed for content of eighteen immune factors (IFN-α, IFN-β, IFN-γ, IFN-ω, IL-2, IL-29, IL-6, IL-8, LIF, TNF-α, CXCL9, CXCL10, CXCL11, CX3CL1, Perforin, Granzyme B, PD-1, TIM3) using a Human Custom ProcartaPlex 18-plex beads array from Invitrogen (ThermoFisher Scientific, Bender MedSystems GmbH, Vienna, Austria) according to the manufacturer’s instructions. In one experiment, PBMC from two HSV-infected donors were exposed to 2 μg/ml Anifrolumab (Saphnelo^®^, Astrazeneca, kind gift from Professor Sören Paludan) or isotype control (Ultra-LEAF™ Purified Human IgG1 Isotype Ctrl Recombinant, Biosite) 60 min before and continuously during incubation with HSV-2 or UV-inactivated HSV-2. Total RNA was isolated from cells after 6 hours using RNase Mini kit (74106, Qiagen). TaqMan RNA-to C_t_ *1-Step* kit, and Taqman probes (Life Technologies) were used to measure the mRNA expression levels of IFNγ (Hs00989291), IFNα2 (Hs00265051), IFNβ1 (Hs01077958) and TATA-box binding protein (TBP) (Hs00427620).

### 2.5 Statistics

Multivariate analyses of pattern recognition “Orthogonal Projections to Latent Structures by means of Partial Least Squares Discriminant Analysis” (OPLS-DA) were performed using the SIMCA-P (version 15.0.2) statistical package (MKS Data Analytics Solutions, Malmö, Sweden). The quality of the models was evaluated by their explanatory power (R2Y) and robustness (Q2Y). Mann Whitney signed rank test was used to compare continuous data and Fisher´s exact test was used to compare categorical data comparing HSV-2 meningitis and HSV-2 genital herpes patients. Kruskal-Wallis one-way ANOVA non-parametric test, with Dunn’s post-test, was used to compare cytokine secretion patterns in PBMC from HSV-2-infected patients in response to different stimuli, two-way ANOVA with Šidák’s multiple comparison test was used to compare the relative mRNA levels of IFNα2, IFNβ1 and IFNγ in PBMC treated with Anifrolumab, and simple linear regression was used to analyze the relationship between age of the patients and cytokine secretion by patient PBMC. These different statistical analyses were performed using GraphPad Prism software 10.3.0 (GraphPad, San Diego, CA, US). A P-value <0.05 was statistically significant.

## 3 Results

### 3.1 Study cohort

Eighty-eight patients with a clinical diagnosis of HSV-2 infection were recruited to the study. The median age at diagnosis was 34 years. 73% were women and 55% had been diagnosed with HSV-2 meningitis (of which 50% also had genital and/or sacral herpes) and the remaining 45% with HSV-2 genital herpes only. 34% of the patients were co-infected with HSV-1 at the time of blood sampling and 19% received prophylactic anti-viral treatment (**Table 1**). The HSV-2 meningitis patients were significantly older that the HSV-2 genital herpes patients, both at diagnosis and at time of sampling. However, there was no statistically significant difference between the groups regarding time from diagnosis to sampling (**Table 1**).

### 3.2 Immune responses to HSV-2 antigens and HSV-2 infection in PBMC from HSV-2-infected patients.

**Figure 1:**
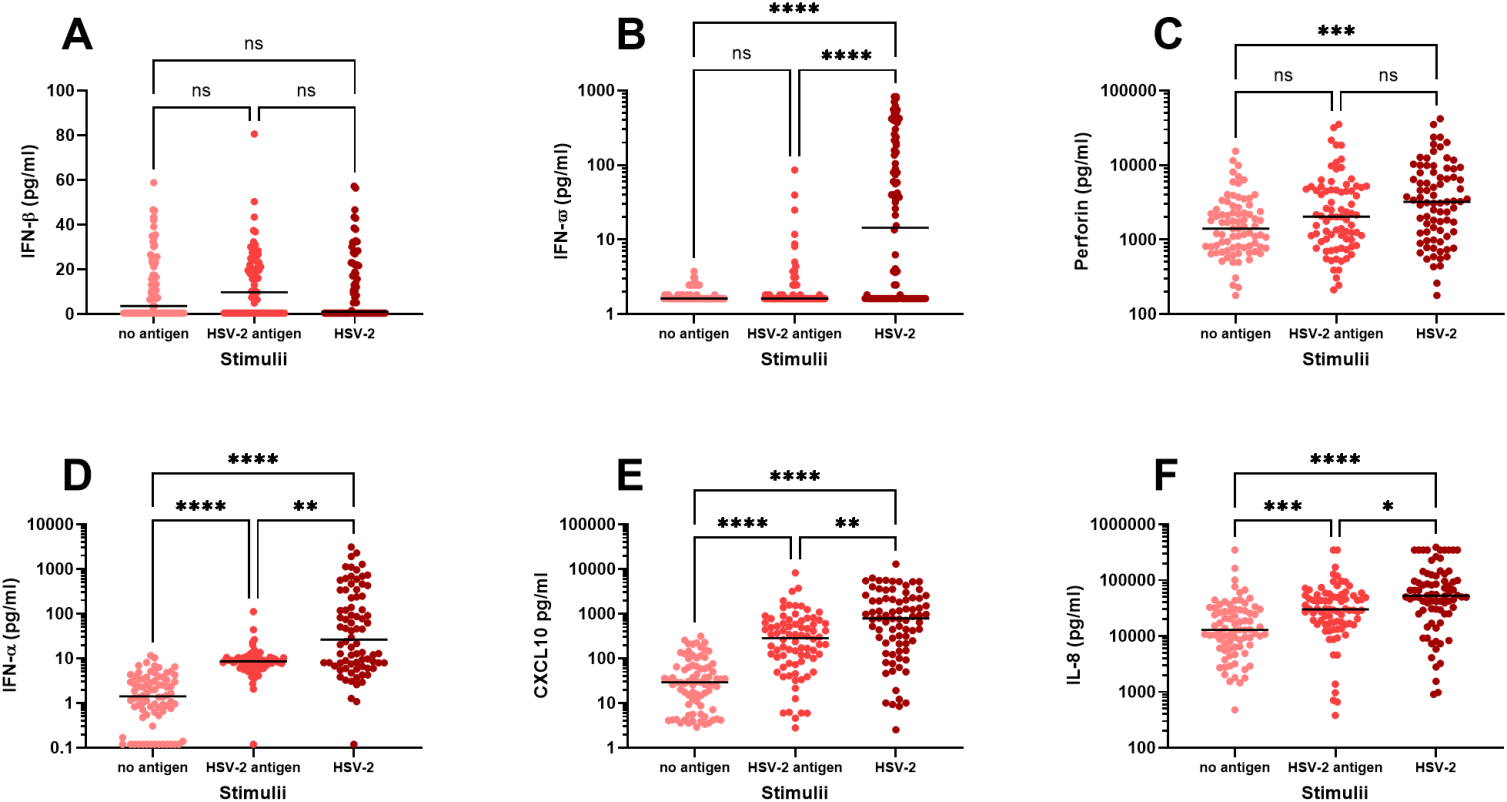
*In vitro* production of immune factors by PBMC from HSV-2-infected individuals. PBMC from HSV-2-infected patients (n=80) were cultured for 48h in the presence or absence of HSV-2 antigen or replication-competent HSV-2 and then analyzed for content of IFN-β **(A)**, IFN-ω **(B)**, Perforin **(C)**, IFN-α **(D)**, CXCL10 **(E)**, IL-8 **(F)** using an 18-plex bead array. *p<0.05, **p<0.01, ***p<0.001, ****p<0.0001 using Kruskal-Wallis one-way ANOVA non-parametric test with Dunn’s post-test.

**Figure 2:**
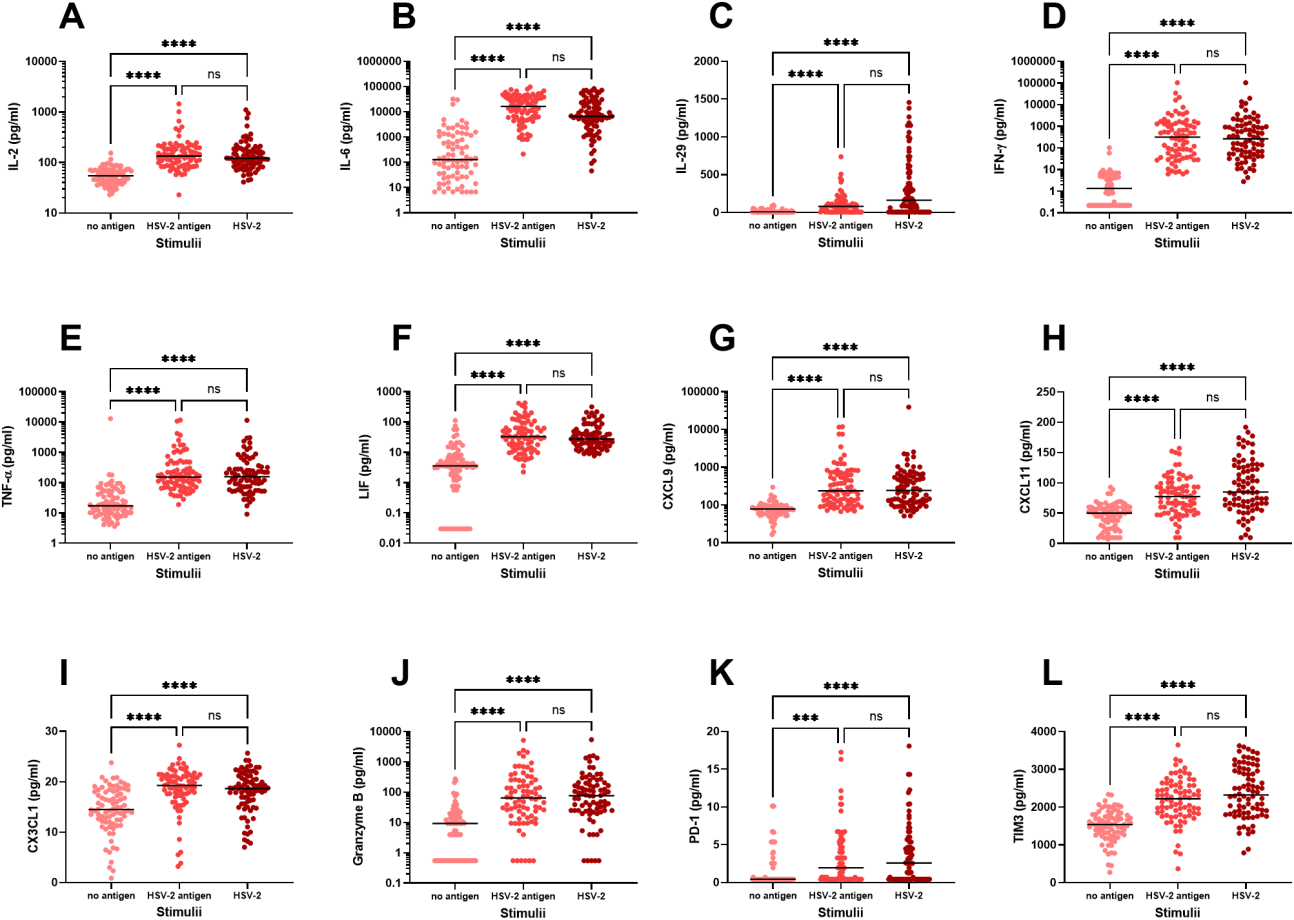
*In vitro* production of immune factors by PBMC from HSV-2-infected individuals. PBMC from HSV-2-infected patients (n=80) were cultured for 48h in the presence or absence of HSV-2 antigen or replication-competent HSV-2 and then analyzed for content of IL-2 **(A)**, IL-6 **(B)**, IL-29 **(C)**, IFN-γ **(D)**, TNF-α **(E)**, LIF **(F)**, CXCL9 **(G)**, CXCL11 **(H)**, CX3CL1 **(I)**, Granzyme B **(J)**, PD-1 **(K)**, TIM3 **(L)**, using an 18-plex bead array. ***p<0.001, ****p<0.0001 using Kruskal-Wallis one-way ANOVA non-parametric test with Dunn’s post-test. Data were missing from 8 patients.

Firstly, we examined the immune activation in PBMC exposed to either UV-inactivated HSV-2 (“HSV-2 antigens”) or to replication-competent HSV-2 (“HSV-2”) and measured the secretion of eighteen immune factors in culture supernatants after 48 hours by multiplex analysis. We observed three distinct response patterns for the different immune factors; i) no upregulation in response to HSV-2, ii) stronger response to replication-competent HSV-2 compared to HSV-2 antigens, and iii) equally strong response to HSV-2 antigens and replication-competent HSV-2. Only one of the 18 immune factors, IFN-β, was not altered after exposure to HSV-2 antigen or to HSV-2 (**Figure 1A**). Five of the immune factors (IFN-α, IFN-ω, CXCL10, IL-8 and perforin) were secreted to a higher degree after exposure to replication-competent HSV-2, indicating that these responses required viral replication (**Figures 1B–F**). The remaining twelve immune factors were induced to a similar degree by HSV-2 antigens and replication-competent HSV-2, indicating that these responses were antigen-specific and did not require viral replication (**Figures 2A–L**).

### 3.3 Demographic and immunological correlates of HSV-2 meningitis

Secondly, we examined if demographic factors (age, sex, prophylactic treatment, HSV-1 co-infection) and immunological parameters (anti-HSV-2 antibody levels, steady-state secretion of cytokines, chemokines and cytotoxic proteins and *de-novo* production of cytokines, chemokines and cytotoxic proteins in response to replicating HSV-2 and to HSV-2 antigens) could be used to distinguish HSV-2 meningitis from HSV-2 genital herpes using a multivariate OPLS-DA model. This approach rendered a stable model (Q2Y=0.47) with an explanatory power of 66% (R2Y=0.66) which indicates that these two patient groups differed significantly from one another (**Supplementary Figure S1A**). Meningitis patients tended to be older and female whereas the patients with genital herpes were more often HSV-1 positive and received prophylactic treatment (**Supplementary Figure S1B**). Meningitis patients had considerably stronger steady-state production of several anti-viral cytokines, chemokines and cytotoxic proteins and had an overall higher *de-novo* production of cytokines and chemokines in response to replicating HSV-2 (**Supplementary Figure S1B**).

**Figure 3:**
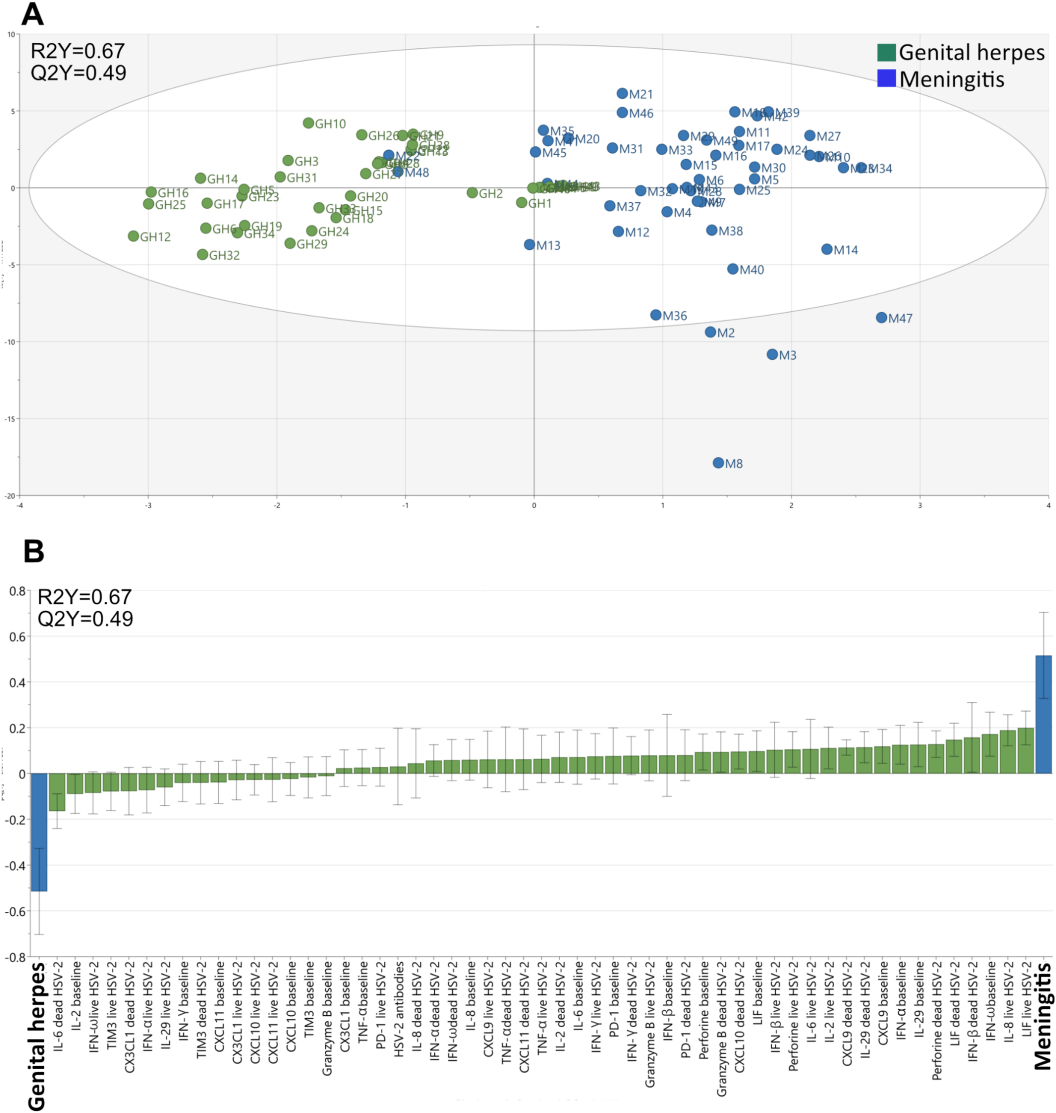
Clustering of individuals with meningitis and genital herpes. The multivariate method “Orthogonal-Projection to Latent Structures Discriminatory Analysis” (OPLS-DA) was used to examine if immunological study parameters (X-variables) could separate individuals with meningitis and genital herpes (Y-variables) based on the secretion of cytokines by PBMC. **(A)** A multivariate model showing the clustering of individuals with meningitis (blue, n = 48) and genital herpes (green, n = 40). The model’s stability (Q) and explanatory power (R) is indicated. **(B)** A loading plot of the OPLS-DA model depicted in *A* shows which of the study parameters that had the largest impact on the separation of the meningitis and genital herpes patients.

To rule out that demographic data dictated the separation of the meningitis and genital herpes cohorts in the OPLS-DA analysis, we performed a second analysis excluding age, sex, prophylactic treatment, and co-infection with HSV-1. Exclusion of demographic data somewhat improved both the stability (Q2Y=0.49) and the explanatory capacity (R2Y=0.67) of the model (**Figure 3A**) indicating that the immune parameters account for the major differences between patients with HSV-2 meningitis and HSV-2 genital herpes. The second model confirm that both the steady-state production of anti-viral cytokines proteins and the *de-novo* production of cytokines and chemokines in response to replicating HSV-2 is higher in meningitis patients (**Figure 3B**). The levels of anti-HSV-2 antibodies had a minor role in distinguishing the two cohorts and did not differ significantly between patients with meningitis and genital herpes in either of the models (**Supplementary Figure S1B**; **Figure 3B**) and thus contributed very little to the separation of the cohorts.

### 3.4 PBMC immune patterns that distinguish HSV-2 meningitis from HSV-2 genital herpes

**Figure 4:**
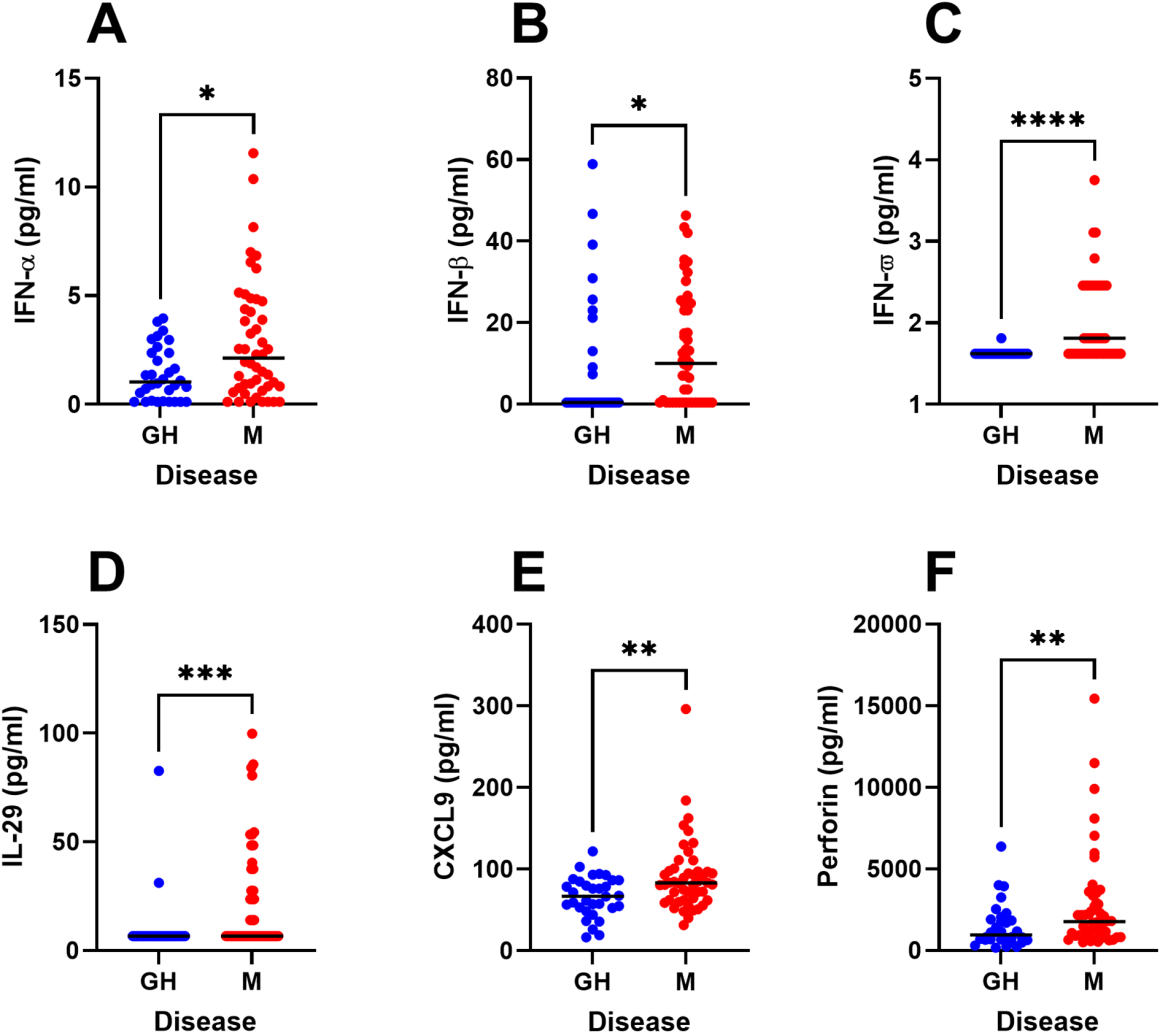
Steady-state production of immune factors in HSV-2 meningitis patients. PBMC from HSV-2 meningitis patients (n=48) and HSV-2 genital herpes patients (n=32) were cultured for 48h in culture media and then analyzed for content of IFN-α **(A)**, IFN-β **(B)**, IFN-ω **(C)**, IL-29 **(D)**, CXCL9 **(E)**, perforin **(F)** using an 18-plex bead array. Mann Whitney signed rank test was used to compare the two groups. *p<0.05, **p<0.01, ***p<0.001, ****p<0.0001. Data were missing from 8 GH patients.

**Figure 5:**
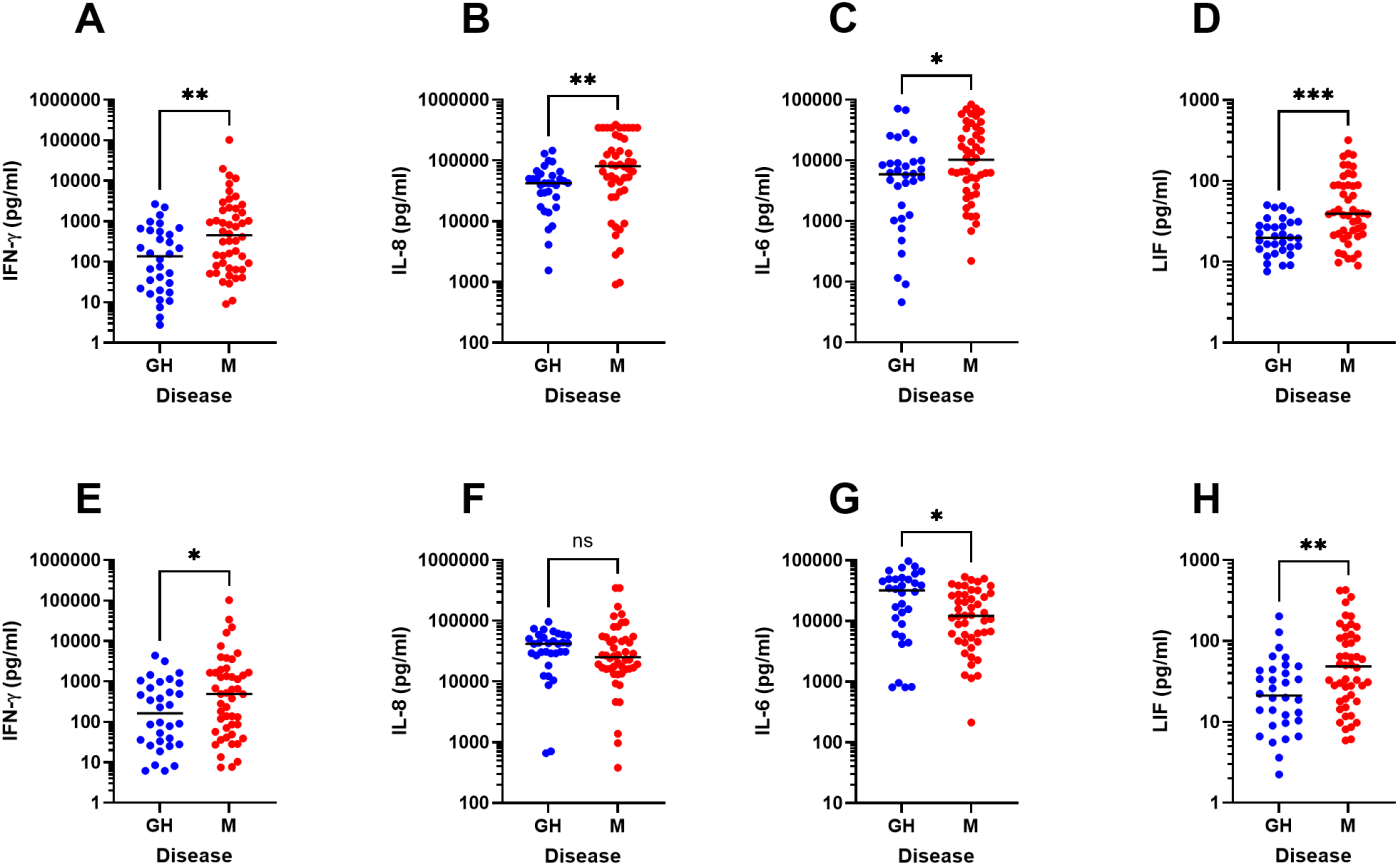
Increased production of immune factors in response to HSV-2 in HSV-2 meningitis patients. PBMC from HSV-2 meningitis patients (n=48) and HSV-2 genital herpes patients (n=32) were exposed to HSV-2 **(A-D)** or HSV-2 antigen **(E-H)** for 48h then analyzed for content of IFN-γ **(A, E)**, IL-8 **(B, F)**, IL-6 **(C, G)**, LIF **(D, H)** using an 18-plex bead array. Mann Whitney signed rank test was used to compare the two groups. *p<0.05, **p<0.01, ***p<0.001. Data were missing from 8 GH patients.

Next, we performed univariate statistical analyses of the major immune parameters that were identified in the OPLS-DA model (**Figure 1B**). We thus confirm that PBMC from HSV-2 meningitis patients spontaneously secrete significantly higher levels of several potent antiviral cytokines (IFN-α, IFN-β, IFN-ω, IL-29, CXCL9, and perforin) during steady state (i.e. in the absence of viral proteins and viral replication) (**Figures 4A–F**) indicating ongoing anti-viral activation of the immune system also during remission. Secondly, PBMC from HSV-2 meningitis patients had significantly higher *de-novo* production of Th1 and inflammatory cytokines (IFN-γ, IL-6, IL-8 and LIF) after exposure to replicating HSV-2 (**Figures 5A–D**) which indicate that PBMC from HSV-2 meningitis patients react to the virus with a stronger inflammatory and recall T-cell mediated immune response than PBMC from patients with HSV-2 genital herpes. The IFN-γ and LIF (but not IL-6 and IL-8) responses to HSV-2 antigen were also higher in PBMC from meningitis patients, indicating that these two cytokines represented recall responses (**Figures 5E–H**).

Given that meningitis patients were older and more often women whereas genital herpes patients were more likely to be HSV-1 seropositive and on anti-viral prophylactic treatment, we also assessed if the levels of cytokines produced differed with age, gender, HSV-1 co-infection and prophylactic treatment. PBMC from females spontaneously secreted significantly higher levels of IL-29 compared to PBMC from men (**Supplementary Figure S2**), while there were no statistically significant sex-related differences in the spontaneous secretion of IFN-α, IFN-β, IFN-ω, CXCL9, or perforin, nor in the *de-novo* production of IFN-γ, IL-6, IL-8 or LIF (data not shown). The only statistically significant age-related difference in cytokine production was spontaneous secretion of IFN-β, which degreased with increasing age (p=0.043). There were no significant differences in the production of any of these cytokines between patients that did, or did not, receive prophylactic treatment, nor did the cytokine secretion patterns differ between patients that were, or were not, HSV-1 seropositive (data not shown).

**Figure 6:**
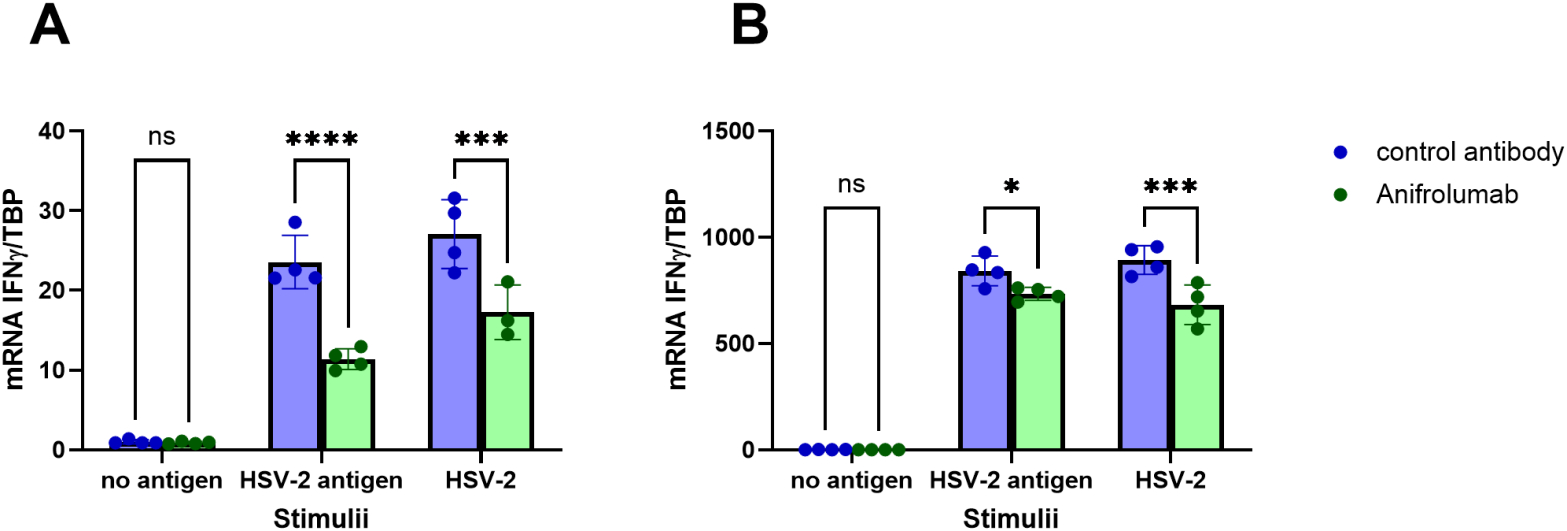
HSV-2-specific IFN-γ-production by PBMC is reduced in the absence of type I IFN receptor signaling. PBMC from two HSV-positive individuals **(A, B)** were treated with Anifrolumab (which binds to the type I IFN receptor) or isotype control in the presence or absence of HSV-2 antigen or replication-competent HSV-2. Relative levels of IFNγ mRNA was assessed after 6 hours by PCR. Two-way ANOVA with Šidák’s multiple comparisons test was used to compare the relative mRNA levels of IFNγ in cells treated with Anifrolumab or with isotype control. *p<0.05, ***p<0.001, ****p<0,0001.

To establish if there is a possible functional connection between the enhanced *de-novo* production of type I IFNs in HSV-2 meningitis patients and the enhanced HSV-2-induced production of IFN-γ, we blocked signaling through the type I IFN receptor in PBMC using Anifrolumab and then exposed the cells to HSV-2 antigen or replication-competent HSV-2 and measured the transcription levels of IFN-α, IFN-β and IFN-γ. As expected, Anifrolumab-treatment significantly reduced the mRNA levels of both IFN-α and IFN-β (**Supplementary Figure S3**). Anifrolumab also significantly reduced the transcription levels of IFN-γ by PBMC in response to HSV-2 antigen and replication-competent HSV-2 (**Figure 6**), indicating that signaling though the type I IFN receptor promote recall IFN-γ production.

### 3.5 Presence of HSV-2 DNA in plasma

To assess if the enhanced spontaneous production of type I IFNs by PBMC from meningitis patients reflected ongoing viral exposure, we measured presence of HSV-2 DNA in plasma from the meningitis patients. However, none of the convalescent meningitis patients had any detectable HSV-2 DNA in their plasma (data not shown).

## 4 Discussion

In this study we show that HSV-2 meningitis patients and patients with HSV-2 genital herpes can be separated based on the production of immune factors by patient PBMC. HSV-2 meningitis patients produced significantly higher levels of innate anti-viral immune factors at steady-state and presented with an overall stronger *in vitro* immune response to the virus.

There were more women than men included in our study cohort which is in accordance with the higher HSV-2 seroprevalence in women compared to men (16) and the higher risk in women of developing symptomatic HSV-2 disease (17). This was particularly evident in the meningitis group where more than 80% of the included patients were women, which is similar to the overall proportion of women among patients with HSV-2 meningitis reported in a recent nationwide Danish cohort study (18). The mean age of diagnosis was 29 and 37 years-of-age for genital herpes and meningitis patients, respectively, which is similar to what has been observed previously in Scandinavia (18, 19). We also confirm that co-infection with HSV-1 is lower among meningitis patients then among patients with genital herpes, although it was higher than previously reported in HSV-2 genital herpes patient cohorts (20, 21). 50% of HSV-2 meningitis patients also had genital and/or sacral HSV-2 manifestations, which is in line with what has previously been observed (21, 22). Thus, our study cohort is representative for HSV-2 genital herpes and HSV-2 meningitis patients except perhaps the high number of recurrencies among the HSV-2 genital herpes patients, most likely because these were highly symptomatic patients attending a university hospital clinic rather than primary care.

PBMC from the HSV-2-infected patients produced elevated levels of 17 out of the 18 immune factors analyzed. The exception was IFN-β. We have previously detected HSV-2-induced production of IFN-β *in vitro* in PBMC from HSV-2-infected patients using ELISA, but these responses were documented at a considerable earlier time-point post viral exposure (18 hours) (23), and the IFN-β might thus have waned by the 48 hours’ time-point used in the current study. The other type I IFNs measured, IFN-α and IFN-ω, were as expected produced in considerable amounts by PBMC from HSV-2-infected patients in response to replication-competent HSV-2. Most other immune factors were induced equally well by HSV-2 antigens and replication-competent HSV-2 indicating that these responses were mainly T-cell derived recall responses, for example the classical CD4+ T-cell cytokines IL-2, IL-6 and IFN-γ, the CD8+ T-cell cytotoxic proteins perforin and Granzyme B, as well as chemokines that attract and activate T-cells, i.e. CXCL9, CXCL10 and CXCL11.

OPLS-DA analysis is used to discriminate between two groups. Using this method, we were able to separate the HSV-2 meningitis and the HSV-2 genital herpes cohorts based on immune factors. Meningitis patients were distinguished by the spontaneous production of several anti-viral immune factors by PBMC including type I and type III IFNs. Age, gender and prophylactic anti-viral treatment had no impact on the production of these immune factors, despite the difference in age, sex and anti-viral prophylactic treatment between HSV-2 meningitis and HSV-2 genital herpes patients. The enhanced spontaneous production of anti-viral immune factors was not associated with a sustained systemic presence of HSV-2 as no viral DNA could be detected in patient plasma. Instead, the spontaneous production of IFN-α, IFN-β, IFN-ω, IL-29, CXCL9, and perforin could imply that HSV-2 meningitis generates a more pronounced systemic innate immune response leading to stronger trained innate immunity (24), and epigenetic imprinting (25) than HSV-2 genital herpes.

PBMC from HSV-2 meningitis patients secreted higher levels of several Th1 (IFN-γ) and inflammatory cytokines (IL-6, LIF, IL-8) in response to HSV-2 compared to PBMC from HSV-2 genital herpes patients. We thus confirm previous studies showing enhanced IFN-γ production by PBMC from meningitis patients (8) and show that the HSV-2-induced production of several other cytokines, i.e. IL-6, LIF and IL-8, are produced to a higher degree in PBMC from meningitis patients. We also confirm previous studies showing that the frequency of HSV-2 recurrencies does not affect the magnitude of the virus-specific T-cell cytokine responses (26, 27). It is known from other studies that type I IFN can prime stromal cells for IL-8 production (28), T-cells to produce IFN-γ (29), and macrophages to produce IL-6 (30), and we confirm that type I IFN augments the HSV-2-induced IFN-γ production by PBMC. We thus hypothesize that the increased production of these cytokines in PBMC from HSV-2 meningitis patients reflect a sustained type I IFN priming in this patient group.

We detected no difference in HSV-2-specific antibody levels between patients with HSV-2 meningitis and HSV-2 genital herpes even though HSV-2 meningitis patients are less likely to be co-infected with HSV-1 (21, 31). Thus, the HSV-2 type-specific antibody responses did not differentiate the meningitis and the genital herpes cohorts and was not affected by the presence of anti-HSV-1 antibodies.

In summary, we show that HSV-2 meningitis leads to a more profound activation of both innate and acquired PBMC immune responses, compared to that of HSV-2 genital herpes. Whether these differences in immune activation are the cause, or the consequence, of different disease manifestations remains to be determined.

Limitations of the study: PBMCs are not the ideal sample for comparing HSV-2 meningitis and HSV-2 genital herpes, as they do not directly reflect immune responses in the CNS. Thus, we do not know if the responses in PBMC reflect the immune responses in the CNS, nor to what extent other cells, e.g. tissue-resident microglia, affect the development of different HSV-2 disease manifestations.

## Data Availability

The original contributions presented in the study are included in the article/**Supplementary Material**. Further inquiries can be directed to the corresponding author.
