# Supplementary figures and images for: Cytokine secretion patterns distinguish herpes simplex virus type 2 meningitis from herpes simplex virus type 2 genital herpes
Source: Front Immunol. 2025 Jun 4;16:1515741. doi: 10.3389/fimmu.2025.1515741 (PMC12174149; doi:10.3389/fimmu.2025.1515741)

## Slide 1
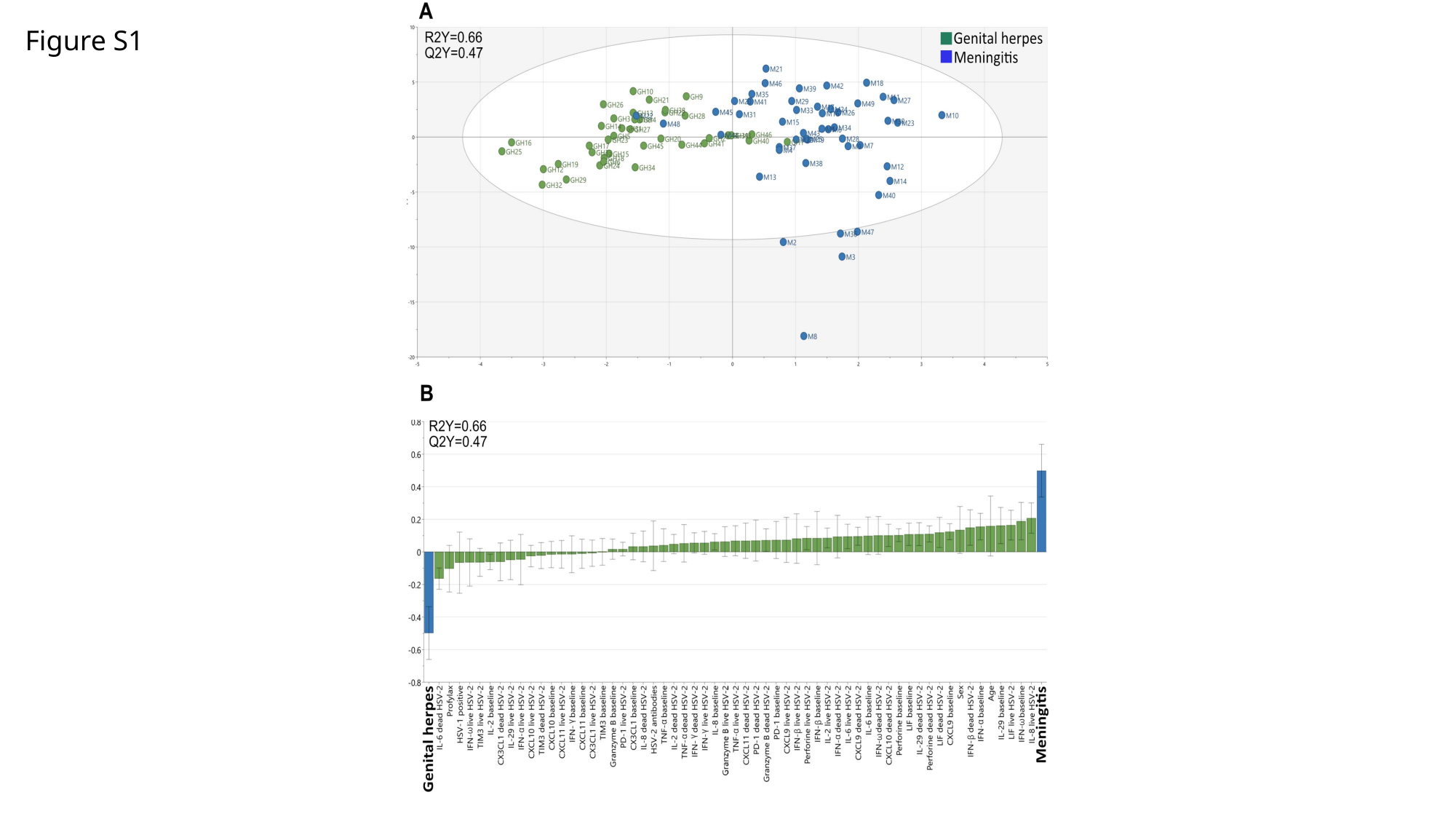

Figure S1

## Slide 2
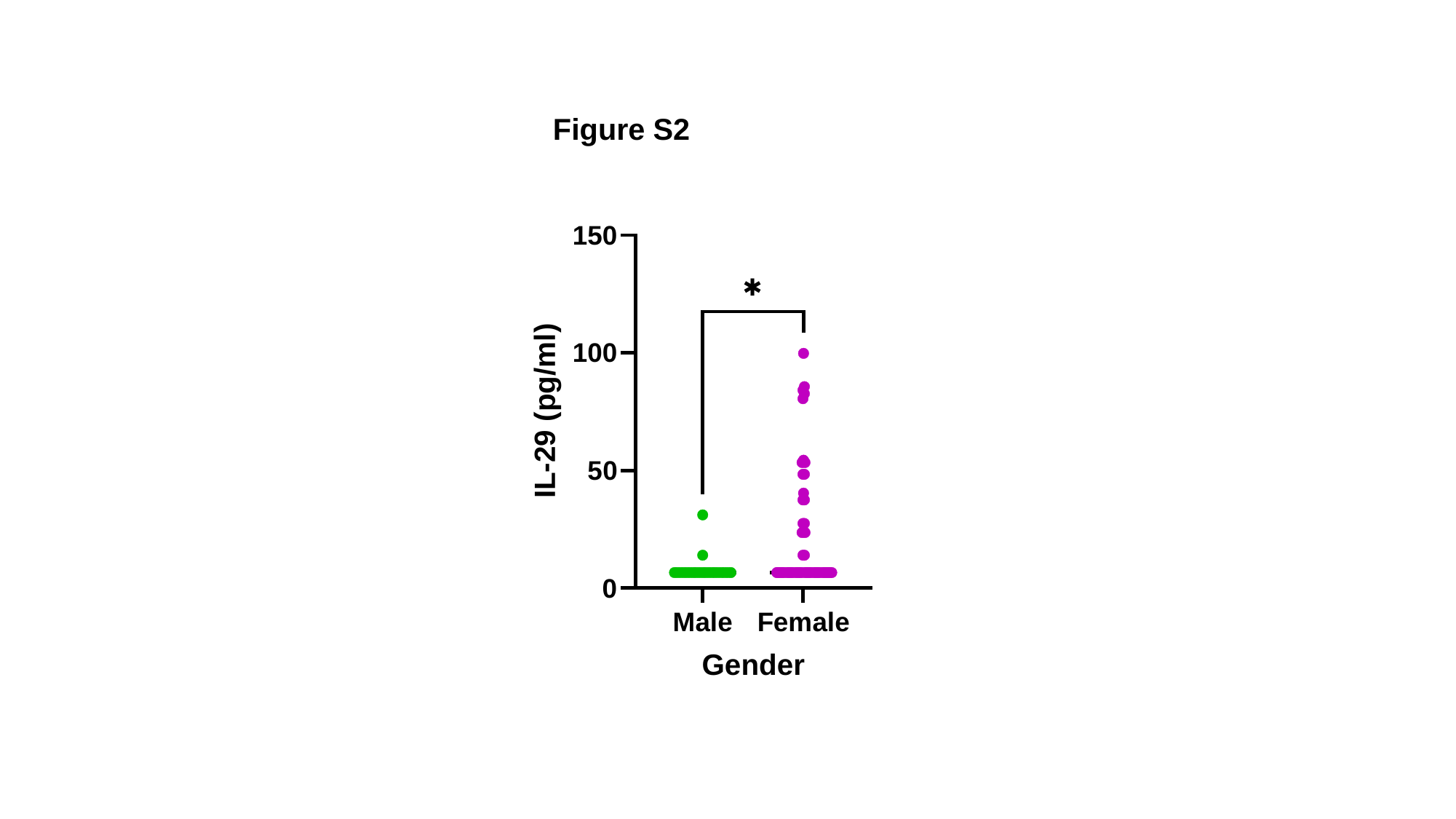

## Slide 3
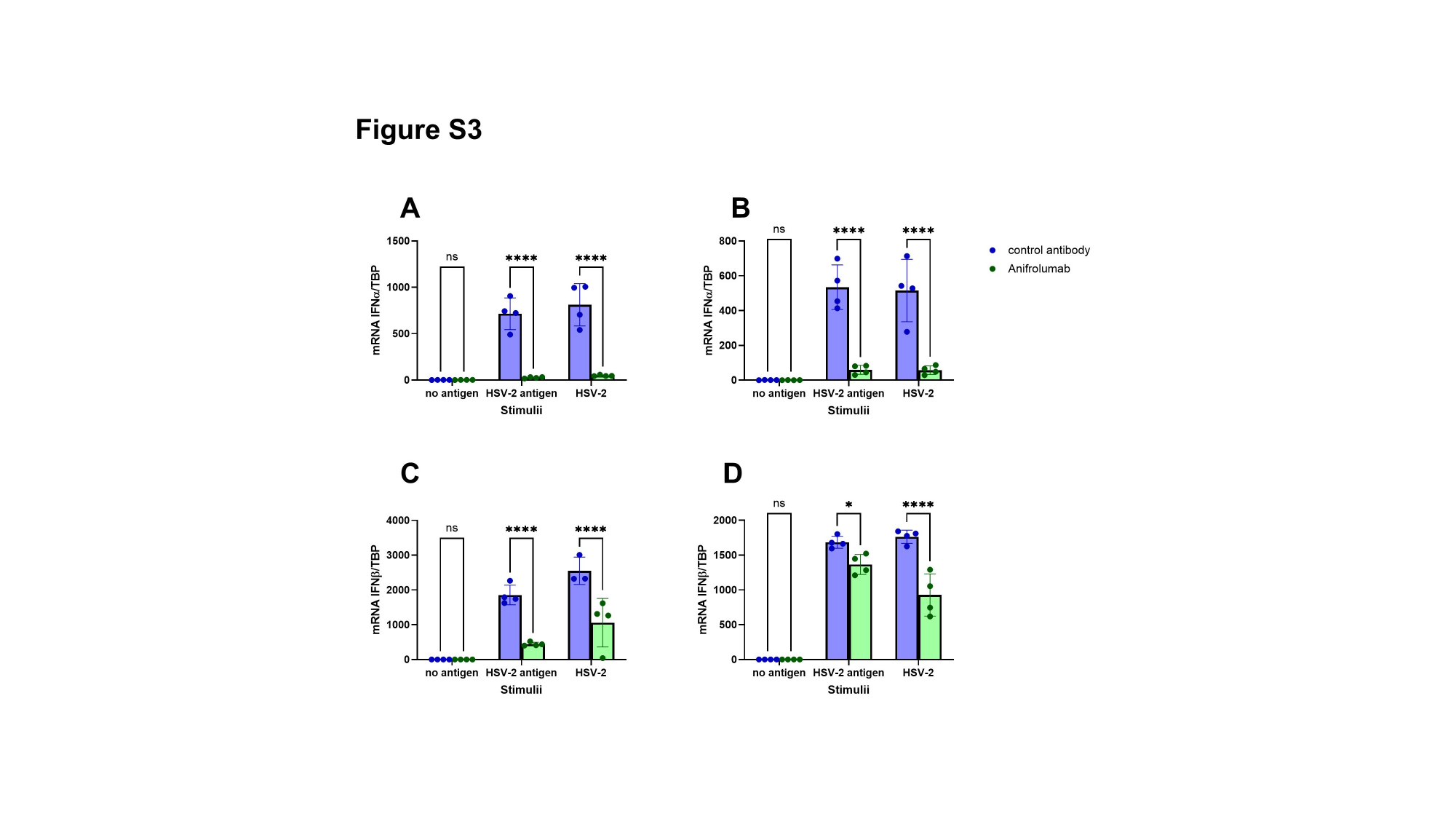

Supplement: Supplementary file 1 [file Presentation1.pptx]
